# THE IMPACTS OF LIFESTYLE ON DEPRESSION AND LIFE SATISFACTION AMONG CHINESE OLDER ADULTS: A 7-YEAR FOLLOW-UP STUDY

**DOI:** 10.1093/geroni/igac059.2714

**Published:** 2022-12-20

**Authors:** Man-Man Peng

**Affiliations:** Beijing Normal University, Zhuhai, Guangdong, China (People’s Republic)

## Abstract

This study aims to explore the long-term effects of lifestyle-related factors and physical health on life satisfaction among older adults by transitions in mental health conditions. Using data derived from the China Health and Retirement Longitudinal Study (CHARLS), the analytic sample included 643 older adults. Linear regression analyses were used to examine the cross-sectional and longitudinal associations of lifestyle-related factors and physical health with depression risk and life satisfaction in older adults. In this study, sleep duration and multimorbidity was found to be significantly related to baseline and follow-up depressive symptoms in older adults. Compared to non-drinkers, current drinkers reported more severe depressive symptoms. More depressive symptoms were associated with worse impairment in physical function or Activities of Daily Living (IADLs). Among older adults remaining no depressive symptoms at baseline and follow-up, current drinkers tended to have lower life satisfaction than non-drinker. Shorter sleep duration showed a longitudinal correlation with lower life satisfaction. In the subgroups of emerging depression, past drinkers tended to have lower life satisfaction than non-drinkers, and baseline multimorbidity significantly predicted lower subsequent life satisfaction. In conclusion, our findings identified drinking and shorter sleep duration as the lifestyle-related detrimental factors of late-life depression and life satisfaction in Chinese community-dwelling older adults. Other physical-health-related risk factors of depression included worse impairment in physical function or IADLs, and multimorbidity. Our findings have implications for future psychosocial interventions targeted at alleviating depressive symptoms and promoting life satisfaction of the older adults based on their long-term mental and physical health conditions.

